# The role of parental health beliefs in seeking an eye examination for their child

**DOI:** 10.1186/s12886-023-02994-2

**Published:** 2023-06-13

**Authors:** Dua Masarwa, Yulia Niazov, Merav Ben Natan, Dina Mostovoy

**Affiliations:** 1 Department of Ophthalmology, Barzilai Medical Center, Ashkelon, Israel; 2 Pat Matthews Academic School of Nursing, Hillel Yaffe Medical Center, Hadera, Israel

**Keywords:** Children, Parents, Eye examination, Health beliefs, Vision problems

## Abstract

**Background:**

We aimed to explore the role of parental health beliefs in parent seeking of eye examinations for their children, using the Health Belief Model.

**Methods:**

In this quantitative correlational survey study, 100 parents who presented to Barzilai University Medical Center in July 2021 to perform an eye examination to their child completed a questionnaire.

**Results:**

Only 29.6% of the parents knew that a vision screening is performed in first grade, and 10% of the parents were unsure about where to find local eye care for their kids. Moreover, 19% of the parents indicated that they were concerned that their child would be prescribed glasses unnecessarily, and 10% believed that wearing glasses would weaken their child’s eyes. Various parental health beliefs regarding children’s eye examinations were found associated with parent seeking of eye examinations for their child. Thus, perceived susceptibility (r = 0.52, p < 0.01), perceived benefits (r = 0.39, p < 0.01), and perceived barriers (r=-0.31, p < 0.01) are associated with parent seeking of eye examinations for their child. Also, parents’ level of knowledge was associated with seeking eye examinations for their child (r = 0.20, p < 0.01).

**Conclusion:**

Parent perceptions of the child’s susceptibility to vision problems and perceived barriers to seeking eye examinations predicted parents seeking of eye examinations for their child. Interventions aimed at increasing timely eye examinations among children should focus on raising parent awareness of vision problems in childhood, dispelling misconceptions, and providing parents with practical information regarding available services.

**Supplementary Information:**

The online version contains supplementary material available at 10.1186/s12886-023-02994-2.

## Background

Normal vision development is crucial for optimal childhood development lifelong functioning [1]. Unfortunately, decreased vision in young children is prevalent [2–5]. It has been reported that 24% of children in Israel are at risk of vision problems [6].

Causes of decreased vision include refractive error, strabismus, media opacities, etc. These conditions cause abnormal visual stimulation, resulting in amblyopia, a significant cause of visual impairment. Amblyopia develops due to visual system insult during the critical [7] development period due to an ocular pathology that interferes with visual cortical development, affecting approximately 4.3% of children [8].

Most of the vision problems in children are asymptomatic; hence it is difficult for parents to identify them, and pediatric vision screening is of paramount importance. Timely identification of vision problems in children is crucial for timely treatment and prevention of amblyopia, which may be irreversible [9–11].

Many professional groups advise newborns to have their vision checked. For instance, the American Academy of Ophthalmology advises screening between the ages of six months and one year [6], whereas the American Association for Pediatric Ophthalmology and Strabismus advises performing vision screening at least once between the ages of one year and three years. The risk-benefit analysis of screening at this age was not supported by the literature, according to the United States Preventive Services Task Force (USPSTF), an independent group of experts in evidence-based preventive medicine. They came to the conclusion that no screening methods have been shown to be successful for children under the age of three and that the available data is insufficient to determine the net benefit of screening this age group. Additionally, children under the age of three frequently struggle to participate during testing, resulting in either false-positive results or incomplete screenings. Consequently, the USPSTF concluded that 3–5 is the ideal age for vision screening [12].

In Israel the national preventive health services funded by the government include visual acuity screening performed at ages 3 and 5 at community-based mother and child health clinics and in the first grade at school. However, visual acuity screening, only assesses central visual acuity; it does not check for other vision issues such strabismus, astigmatism, refractive errors, etc.; it may also fail to detect amblyopia [6].

Despite the importance of timely vision screening and treatment, many children are not screened [13]. As children rely upon their caregivers, usually the parents, to gain access to care, so it is crucial to understand what drives parents to seek eye care for their children. There is evidence that parental perceptions play a role in parents seeking eye care for their children [14–16]. Moreover, eye screening for children is not effective when there is no cooperation from the parents for further treatment [14, 15]. Studies have shown that parental perceptions may prevent them from seeking the necessary eye care. These include concerns about the treatment, previous family history with similar conditions that had no encouraging solution, disagreement between the parents about eye examination needs, and more. The cost of eye care services, poverty, access to eye care, and other logistical issues, including taking time off from work and long waiting periods at the clinic, also play a role [13, 16]. In a study that examined parents’ knowledge, attitudes, and practices regarding pediatric eye medical services in the Aseer region of Saudi Arabia, it was discovered that parents were aware of pediatric eye health; however, worries about the screening results prevented them from seeking eye care for their children [17]. In Swaziland a developing country, parents also don’t understand the value of routine eye exams for their kids or how to provide them with basic pediatric eye care [18]. In addition, parental misconceptions regarding eye care for children include that the suggestion that the child is too young for the eye test, that eyeglasses will make his eyes weaker, or that he will be given eyeglasses he does not need, and more, which prevent seeking eye care [19]. Bruce et al. [20] found that the combination of parents’ perceptions influenced parental decisions regarding the child’s attendance to vision screening and the decision to adhere to utilization of eyeglasses, such as the perceived severity of the visual reduction and the perceived benefit of eyeglasses [20]. However, the relative contribution of the various parental perceptions to parent seeking of eye care for their child is unclear.

For decades, the Health Belief Model (HBM) has been one of the most widely used conceptual frameworks for explaining the changing and maintenance of health-related behavior. The model provides an explanatory framework for the adoption of health-related behavior. It includes perceived susceptibility, that is, an individual’s assessment of their chances of developing a condition; perceived severity, that is, an individual’s opinion as to the seriousness of the condition; perceived benefits, that is, an individual’s opinion as to whether a new behavior is better than current behavior; and perceived barriers, that is, an individual’s opinion as to what will prevent them from adopting a new behavior. The model also refers to cues to action, that is, factors that will prompt a person into changing behavior [21].

The present study aimed to explore the role of parental health beliefs in parent seeking of eye examinations for their children at a pediatric ophthalmology clinic using HBM.

## Methods

This is a quantitative correlational survey study conducted at Barzilai University Medical Center, Ashkelon, Israel. The Barzilai Medical Center in Israel offers vision screening services for children up to age 5. The tests include near visual acuity test, stereotest, eye movement test, and squint assessment. For the near visual acuity test, the Jaeger near acuity chart with LEA symbols was used. All parents who presented with their child to the Medical Center during July 2021 for any purpose were asked if they would like to have their child given an eye examination. Those who agreed were then asked to complete a questionnaire (Appendix 1). To achieve a 95% confidence interval with a 50% anticipated frequency of knowledge and 15% acceptable error margin, we needed at least 150 participants, based on research by Donaldson et al. [19]. During this month, 150 eye examinations were performed to 150 children. One hundred parents consented to take part in the study and completed a questionnaire, for a response rate of 67%.

The questionnaire was informed by the HBM and was based on the questionnaire designed by Donaldson et al. [19]. It explored parental health beliefs, consisting of four subsections as follows: perceived susceptibility (2 items, e.g., ‘’I have concerns that my child may be at risk of a vision problem’’), perceived severity (2 items, e.g., ‘’I fear that a vision problem will affect my child’s development’’), perceived benefits (2 items, e.g., ‘’Good vision is important for proper childhood development’’), and perceived barriers (10 items, e.g., ‘’Wearing eyeglasses may weaken my child’s eyes’’). The participants were asked to state the level of their agreement with the various statements in these four subsections on a Likert scale ranging from 1–6, where 1 – ‘’strongly disagree’’ and 6 – ‘’strongly agree’’.

In addition, the participants were asked to note the reasons for seeking an eye examination for their child (cues to action) (10 items, e.g., ‘’Advised by a healthcare provider or a teacher’’). The participants were also asked about their previous vision screenings. Moreover, the questionnaire included knowledge questions regarding vision and eye examinations in children (4 items, e.g., ‘’Children can undergo an eye examination only when they are familiar with numbers and letters”). For each correct answer the respondent received one point. The knowledge variable was calculated as a sum of the points, with a maximum score of 4 points (one point for each correct answer).

Finally, the questionnaire collected sociodemographic data, including parental sex, age, education, and religion, number of children, and the tested child’s position among other siblings.

Statistical analysis. In the current study, the internal reliability of the questionnaire’s components ranged from 0.82 to 0.89. The back-and-forth translation technique was used to translate the questionnaire into Hebrew. Three subject matter experts examined it and determined it to be reliable. In order to capture the key components of knowledge, the knowledge items were devised in conjunction with ophthalmology experts.

### Statistical analysis

Statistical analysis was performed using SPSS software for Windows, version 26 (SPSS, Chicago, IL, USA). The normality of the data distribution was tested using the Kolmogorov–Smirnov test. All variables had a normal distribution, therefore descriptive statistics – percentages, means, and standard deviations (SD) – were used to describe the research data. Pearson’s correlations were conducted to explore the associations between the research variables.

Finally, multiple linear regressions were used to explore the association between the predictors of parents’ seeking eye examinations for their child and explanatory variables found to be associated: perceived susceptibility of the child to vision problems, perceived barriers to seeking an eye examination for the child, level of knowledge regarding vision and eye examination in children, and perceived benefits of eye examinations in children. A significance level of p < 0.05 was considered statistically significant for all analyses.

## Results

One hundred parents consented to participate in the study (74 mothers and 26 fathers) and completed the questionnaire. Parents’ average age was 35.86 ± 0.90 (range 22–56). They had, on average, 3.14 ± 1.84 children. Most parents were Jewish (94%), while a minority were Arabs (6%). Approximately one third defined themselves as secular (36%), 24% religious, and 30% traditional. Approximately two-thirds of the parents had an academic education (66.3%).

Approximately two-thirds of the parents (68%) were concerned about their child’s vision. The reasons that the parents sought an eye examination for their child are presented in Table 1. The three most frequent reasons were: concerns about poor vision, eyes not being straight or having a turn, and being advised by a healthcare provider or teacher.

**Table 1:** Parental reasons for seeking eye care for their child

Reason for seeking eye care	Percentage
Concerns about poor vision	94%
Concerns about eyes not straight/have a turn	88%
Advised by healthcare provider or teacher	87%
Complaints of double vision	81%
Routine check up	70%
Family history	65.3%
Headaches	57.6%
Poor concentration/short attention span	57%
Poor school achievement and/or difficulties with literacy	54%

Parents’ perceived barriers to seeking an eye examination for their child had a low mean (1.45 ± 0.74, on a scale of 1–6, maximum six). However, 19% of the parents indicated that they were concerned that their child would be prescribed eyeglasses unnecessarily. In addition, 10% of the parents believed that wearing eyeglasses would weaken their child’s eyes. Moreover, 10% of the parents were unsure about where to find local eye care for their kids. Of note, only 29.6% of the parents knew that vision screening is performed in first grade as part of school health services funded by the national government.

Parents had a moderate level of knowledge regarding vision and eye examinations in children (2.20 ± 1.42 Max 4 points); 77% knew that it is possible to perform an eye examination in children who have not yet learned numbers and letters, 60% knew that school vision screening does not check all vision problems, and 55% knew that intermittent squinting between ages 1–7 is not normal. However, only 28% of the parents knew that wearing glasses under age 7, when necessary, strengthens vision.

Various parental health beliefs were associated with parent seeking of eye examinations for their child. Thus, parental perceptions of the child’s susceptibility to vision problems (r = 0.52, p < 0.01), perceptions of eye examinations in children (r = 0.39, p < 0.01), and perceived barriers to seeking eye examinations (r=-0.31, p < 0.01) were found associated with parent seeking of eye examinations for their child.

In addition, an association was found between parents’ level of knowledge regarding vision and eye examinations in children and parent seeking of eye examinations for their child (r = 0.20, p < 0.01).

Results of the multiple linear regression analysis revealed that parent perceptions of the child’s susceptibility to vision problems and perceived barriers to seeking eye examinations predicted parents seeking of eye examinations for their child, explaining adjusted R^2^ = 28% of the variance in the phenomenon under study [F(4,95) = 7.40, < 0.01]. Of note, the child’s perceived susceptibility to vision problems is a more significant factor than perceived barriers to seeking eye examinations (Table 2).

**Table 2:** Multiple linear regression analysis of predictors of parent seeking of eye care for their child

Variable	B	S.E.	β	P-value
Perceived susceptibility of the child to vision problems	0.79	0.21	0.45	0.01
Perceived barriers to seeking an eye examination for the child	0.57-	0.28	0.19-	0.04
Level of knowledge regarding vision and eye examination in children	0.12	0.16	0.08	0.44
Perceived benefits of eye examinations in children	0.05-	0.24	0.03-	0.84

B = unstandardized beta; S.E = standard error; β = standardized beta; P-value = probability value

## Discussion

The present study explored the role of parental health beliefs in seeking eye examinations for children. In this study, parents’ perceived barriers to seeking an eye examination for their child had a low mean. However, unawareness and various misconceptions were evident. Thus, only 29.6% of the parents were aware of the vision screening performed in first grade as part of the school health services funded by the national government. Unawareness of these services may lead to unawareness of the screening outcome, preventing parents from seeking further care in case of failed screening [22, 23]. Hence. instruction and communication with the parents regarding screening timing and results is clearly needed.

Moreover, in our study, approximately two-thirds of the parents were concerned about their child’s vision. However, 10% of the parents were unsure about where to find local eye care for their kids. Similar statistics were reported in a UK study [18]. As to various parental misconceptions, in the present study 19% of the parents indicated that they were concerned that their child would be prescribed eyeglasses unnecessarily. In addition, 10% of the parents believed that wearing eyeglasses would weaken their child’s eyes. Similar findings were reported in previous studies [14, 15]. Correcting these misconceptions nay lead to improved parent responsiveness to eye care.

The present study also revealed knowledge deficits among the parents regarding vision and eye examinations in children. Only 60% knew that the vision screening conducted at school does not check all vision problems, and only 55% knew that intermittent squinting between ages 1–7 is not normal. Moreover, only 28% of the parents knew that wearing eyeglasses under age 7, when necessary, strengthens vision. Knowledge deficits may be an additional barrier to parent seeking of eye care for their children. Therefore, it is mandatory to carry out parental education, point out what is not normal in a child, and form awareness of screening timing and results even if there are no suspicious findings.

It should be noted that a large proportion of parents who took part in this study were well-educated. Therefore, the rate of unawareness, misconceptions, and knowledge deficits might be much higher in the general population. The present study revealed that parental health beliefs, namely perceived susceptibility of the child to vision problems, perceived benefits of eye examinations in children, perceived barriers to seeking eye examinations, and knowledge regarding vision and eye examinations in children, are associated with parent seeking of eye examinations for their child. These findings suggest that health beliefs are interlinked in parental decision-making regarding eye care for their child, thus supporting previous studies [18, 24].

In this study, the child’s perceived susceptibility to vision problems and parents’ perceived barriers to seeking an eye examination for children predicted parent seeking of eye examinations for their child. These findings support previous studies that identified perceived barriers as an essential factor preventing parents from seeking eye care for their child [19, 20] and introduce parent perceptions of the child’s susceptibility to vision problems as another important factor that might explain what prompts parents to seek eye care for their child.

Finally, in the present study the three leading reasons that parents sought an eye examination for their child were concerns about poor vision, concerns about eyes not being straight or having a turn and being advised by a healthcare provider or teacher. Interestingly, the reasons reported in the present study, including the least popular reasons, are similar to those reported in a UK study by Donaldson et al. [19] suggesting that parental decision-making regarding eye care of their child in different countries may be similar.

This study has several limitations. Thus, convenience sampling limits the generalizability of the research results. Namely, generalizability may be limited to parents from the Jewish population who have an academic education. In addition, a particular bias cannot be excluded, as parents who took part in this study already expressed interest in taking their children to an eye examination. However, the consistency of our findings with previous studies supports the validity of the research results. Finally, a cause-and-effect relationship cannot be established due to the research design.

## Conclusions

The study revealed that health beliefs play an essential role in parent seeking of eye care for their children. Namely, parents will seek an eye examination for their child if they believe that their child is susceptible to vision problems, are free of misconceptions, have adequate knowledge regarding vision and eye examinations in children, and are aware of available services. Thus, interventions that aim to improve parental education about eye care and examination timing, while raising awareness regarding childhood vision problems, dispelling misconceptions, and providing parents with practical information regarding available services, are needed. It also seems that national public health messaging is needed to reach as many parents as possible.

## Electronic supplementary material

Below is the link to the electronic supplementary material.

Supplementary Material 1

## Data Availability

The data that support the findings of this study are available from the corresponding author, Dr. Merav Ben Natan, upon reasonable request.
