# Supplementary material for: The role of parental health beliefs in seeking an eye examination for their child
Source: BMC Ophthalmol. 2023 Jun 13;23:269. doi: 10.1186/s12886-023-02994-2 (PMC10262523; doi:10.1186/s12886-023-02994-2)
Supplement: Supplementary file 1 — Supplementary Material 1 [file 12886_2023_2994_MOESM1_ESM.docx]

**Appendix**

**The research questionnaire**

**Dear parent!**

We are conducting a study that explores factors associated with parental seeking of eye examinations for their children. We would be grateful if you would answer all the questions, to maximize the benefit of this questionnaire.

The questionnaire is anonymous and the data will be used for research purposes only.

Thank you in advance,

The research team

**Please circle the number as appropriate:**

| 2 –  Uncertain | 1 – Agree | 0 – Disagree |  |  |
| --- | --- | --- | --- | --- |
| 2 | 1 | 0 | Is a routine vision screening test performed at your child’s school or kindergarten? | 1 |
| 2 | 1 | 0 | Were you ever concerned that your child may have a vision problem? | 2 |
| 2 | 1 | 0 | If you were concerned about your child's vision, would you know how to check it? | 3 |
| 2 | 1 | 0 | Do any first degree relatives have a vision problem (wear eyeglasses, have a lazy eye, or squint)? | 4 |
| 2 | 1 | 0 | Has your child previously had any eye tests? | 5 |
| 2 | 1 | 0 | If your child has previously had an eye test, please note how many times:_____ | 5.1 |
| 2 | 1 | 0 | My children have regular eye examinations performed by an ophthalmologist | 6 |

**Please choose a number between 1 (strongly disagree) and 6 (strongly agree), that represents the level of your agreement that the following factors may encourage you to take your child for an eye test.**

| 6 | 5 | 4 | 3 | 2 | 1 | Advised by a physician, family healthcare center nurse, or teacher | 7 |
| --- | --- | --- | --- | --- | --- | --- | --- |
| 6 | 5 | 4 | 3 | 2 | 1 | Concerns about poor vision of my child | 8 |
| 6 | 5 | 4 | 3 | 2 | 1 | Concerns about my child's eyes not being straight/having a turn | 9 |
| 6 | 5 | 4 | 3 | 2 | 1 | My child suffers from headaches | 10 |
| 6 | 5 | 4 | 3 | 2 | 1 | Poor concentration/short attention span of my child | 11 |
| 6 | 5 | 4 | 3 | 2 | 1 | Poor school achievement of my child and/or difficulty reading | 12 |
| 6 | 5 | 4 | 3 | 2 | 1 | My child’s complaint of double vision | 13 |
| 6 | 5 | 4 | 3 | 2 | 1 | The eye test is a routine check up | 14 |
| 6 | 5 | 4 | 3 | 2 | 1 | Family history of eye problems | 15 |
| 6 | 5 | 4 | 3 | 2 | 1 | Other_____________________________________ | 16 |

**Please choose a number between 1 (strongly disagree) and 6 (strongly agree), that represents the level of your agreement that the following factors may prevent you from taking your child for an eye test?**

| 6 | 5 | 4 | 3 | 2 | 1 | I don't know how or where to schedule an eye test for my child | 17 |
| --- | --- | --- | --- | --- | --- | --- | --- |
| 6 | 5 | 4 | 3 | 2 | 1 | I am worried about the cost of an eye test | 18 |
| 6 | 5 | 4 | 3 | 2 | 1 | I am worried about the cost of eyeglasses | 19 |
| 6 | 5 | 4 | 3 | 2 | 1 | I think my child is too young to have an eye test | 20 |
| 6 | 5 | 4 | 3 | 2 | 1 | I am worried that my child does not yet know all the letters | 21 |
| 6 | 5 | 4 | 3 | 2 | 1 | I don't have time to take my child to an eye test | 22 |
| 6 | 5 | 4 | 3 | 2 | 1 | I do not want my child to wear eyeglasses | 23 |
| 6 | 5 | 4 | 3 | 2 | 1 | I am worried that my child may be given glasses although he/she may not need them | 24 |
| 6 | 5 | 4 | 3 | 2 | 1 | I am worried that if my child is given eyeglasses, it will make his/her eyes weaker | 25 |
| 6 | 5 | 4 | 3 | 2 | 1 | Other_____________________________________ | 26 |

**Please circle whether you agree with the following statements:**

| 2 –  Uncertain | 1 – Agree | 0 – Disagree |  |  |
| --- | --- | --- | --- | --- |
| 2 | 1 | 0 | Children can undergo an eye examination only when they are familiar with numbers and letters | 27 |
| 2 | 1 | 0 | Wearing eyeglasses if you need them when under age 7 will make your eyes and vision stronger | 28 |
| 2 | 1 | 0 | It is normal for a child aged 1–7 to occasionally have an eye turn | 29 |
| 2 | 1 | 0 | School vision screening tests for all eye problems | 30 |

**Please choose a number between 1 (strongly disagree) and 6 (strongly agree), that represents the level of your agreement with the following statements:**

| 6 | 5 | 4 | 3 | 2 | 1 | I have concerns that my child may be at risk of a vision problem | 31 |
| --- | --- | --- | --- | --- | --- | --- | --- |
| 6 | 5 | 4 | 3 | 2 | 1 | Visual impairment can limit employment/driving/daily activities | 32 |
| 6 | 5 | 4 | 3 | 2 | 1 | I fear that a vision problem will affect my child's development | 33 |
| 6 | 5 | 4 | 3 | 2 | 1 | Vision affects children's ability to learn | 34 |

**Personal information:**

35. Gender: 1. Male 2. Female

36. Age:_________________

37. Age of the examined child:_________________

38. The birth order of the examined child in the family:

1. First 2. Second 3. Third 4. Other

39. Gender of the child: 1. Male 2. Female

40. Nationality: 1. Jewish 2. Arab

41. Level of religiosity: 1. Secular 2. Traditional 3. Religious

42. Marital status: 1. Married 2. Single parent 3. Divorced

43. Number of children:_________________

44. Level of education: 1. Elementary 2. High school 3. Academic

45. Eye test results: Abnormal findings:_________________
